# Radiographic Outcome of Endodontic Treatment of Teeth with Primary Apical Periodontitis: Results from a Postgraduate Clinic

**DOI:** 10.3390/dj13120593

**Published:** 2025-12-11

**Authors:** Pia Titterud Sunde, Erika Giving, Tabish Dilshad, Trude Handal, Dag Ørstavik

**Affiliations:** Department of Endodontics, Institute of Clinical Dentistry, University of Oslo, 0455 Oslo, Norway; e.m.giving@odont.uio.no (E.G.); tabish.dilshad@odont.uio.no (T.D.); trude.handal@odont.uio.no (T.H.); dag.orstavik@odont.uio.no (D.Ø.)

**Keywords:** apical periodontitis, cohort study, single-visit, endodontics, treatment outcomes

## Abstract

**Objective:** The aim of the study was to analyze factors influencing the radiographic outcome of first-time endodontic treatment of teeth with periapical lesions. **Methods:** From March 2008 to October 2022, 804 cases of primary apical periodontitis with radiographically detectable lesions were treated conservatively by postgraduate students at the Department of Endodontics. A total of 437 patients had recall 11–48 months after completion. Post-operative and control radiographs of the teeth were scored by the periapical index (PAI) adjusted to define strict and lenient criteria for success. Patients’ sex and age, the tooth treated, the number of visits, and several tooth- and treatment-related factors were registered and related to radiographic outcomes in bivariate and regression, with actual *p* levels recorded. **Results:** Overall success rate was 68% by strict and 83% by lenient criteria. In binary analyses, a high preoperative PAI score, older age, poorer periodontal status, tooth type (anterior teeth and premolars), and higher number of visits were negatively related to the outcome. Logistic regression analysis of the whole material confirmed an adverse effect on outcome by these factors. In particular, the number of visits (OR = 1.3, *p* = 0.003) and the initial PAI (OR = 1.9, *p* < 0.001) were the strongest predictors of reduced success. **Conclusions:** Outcome of treatment of primary apical periodontitis by postgraduate students was negatively affected by higher preoperative PAI score, higher patients’ age, poorer periodontal status, and higher number of visits for completion. **Clinical Relevance:** This study provides clinically relevant insight into multiple prognostic factors that influence the outcome of primary root canal treatment in teeth with periapical lesions, including patient-related, tooth-related, and procedural variables. The results reflect real-world outcomes in a postgraduate clinical setting and confirm the favorable outcome of single-visit treatments found in randomized studies.

## 1. Introduction

Numerous studies have assessed factors that may influence the outcome of conservative treatment of apical periodontitis [1]. Many of the preoperative, perioperative, and postoperative host- and tooth-specific factors that are readily recorded have not been found to significantly impact results in patient-based, cohort studies, or in randomized clinical tests [2,3,4]. However, preoperative periapical diagnosis strongly influences the outcome [3,5], and tooth type, periodontal status, and age are factors with an uncertain relationship to treatment result [2].

The number of visits is not normally included as a variable in cohort studies of treatment outcomes. Randomized studies have established the principle of single-visit treatment as an adequate procedure [6,7], and systematic reviews [8,9,10] have clearly documented that the procedure is viable and performs similarly to the more conventional concept of applying an interim dressing between appointments. Moreover, a meta-analysis of relevant clinical data found no benefit from a calcium hydroxide dressing [11]. However, controlled trials do not address the effect of number of visits in actual clinical practice. Here, case complexity [12] with time constraints may determine whether a tooth is treated in one or more sessions, and many teeth that could be completed in a single visit are likely to be treated in two or more sessions, possibly improving their chance of success. On the other hand, time constraints may cause the tooth to be poorly cleaned, and the temporary filling is a risk factor for bacterial contamination. Thus, while the dressing may reduce the bacterial load in many cases, an increase in the level of infection may also take place [13,14].

Only a few studies have addressed the impact of the number of sessions in a practice setting with conflicting results [15,16]. Thus, the outcome of single-visit treatments outside of randomized studies in a research setting is largely unknown. The single-visit approach has been accepted but not explicitly promoted in the postgraduate endodontic clinic at the University clinic for more than 15 years. The present study aims to assess the radiographic outcome of root canal treatment in teeth with periapical lesions, with specific attention to the impact of the number of visits in comparison with, and adjusted for, other salient prognostic factors. Hypothesis: The number of treatment visits influences the radiographic healing outcome of teeth with periapical lesions, independent of other prognostic variables.

## 2. Materials and Method

### 2.1. Source of Data

The patient databases at the Department of Endodontics provided treatment details and radiographs for all endodontic treatments. The study was conducted in accordance with the Declaration of Helsinki. The study is part of a project that has been reviewed and accepted by the Regional Committee for Medical and Health Research Ethics in Norway (REK 64996, on 18 December 2019).

### 2.2. Case Selection and Treatment

**Figure 1:**
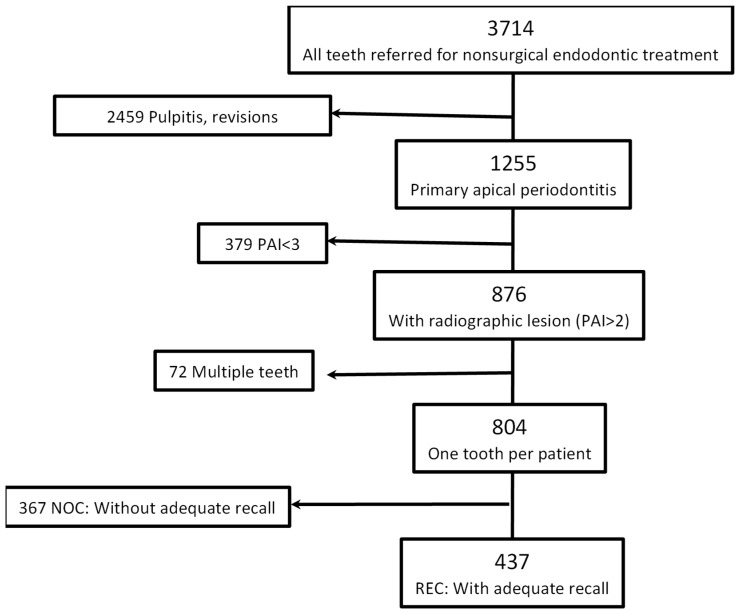
Case selection procedure and definition of groups.

Data for 1255 teeth referred to the postgraduate clinic at the Department of Endodontics for first-time, non-surgical treatment of teeth with periapical lesions between March 2008 and December 2021 were retrieved from the electronic records. Cases with less than 11 months of follow-up data were excluded, as were cases with no or unclear radiographic signs of apical periodontitis (preoperative periapical index [PAI]) score 1 or 2 [17]). If more than one tooth was treated for a patient, only the most distal tooth was included (Figure 1). Table 1 lists the registrations performed for each case. In all, 437 cases fulfilled the inclusion criteria with adequate recall. See Figure 1.

Protocols for non-surgical treatment were stable, with minimal alterations from 2008 through 2021 and followed the Guidelines of the European Society of Endodontology (ESE) [18] and included disinfection of tooth and rubber dam with chlorhexidine, instrumentation by hand, rotary and reciprocating instruments, canal disinfection by sodium hypochlorite, and root filling by AH Plus (Dentsply DeTrey GmbH, Konstanz, Germany) or, in case of open apices or root perforation, TotalFill BC sealer (FKG Dentaire, La Chaux-de-Fonds, Switzerland) Postgraduate students performed all treatments at all levels of the program.

### 2.3. Radiographic Evaluation

The periapical conditions were scored with the PAI scoring system. All scoring was performed by two observers (EG, TD) calibrated against a standard set of 100 periapical radiographs scored twice. Weighted Cohen’s kappa values were 0.8 and 0.71, respectively, which signal substantial agreement [19]. All radiographs were digital, acquired by the Digora phosphor storage plate system (Digora for Windows, Soredex OY, Tuusula, Finland, version 2.5 and newer, Cliniview, Palodex Group OY, Tuusula, Finland) and evaluated on screen in a dimly lit room. PAI scores at the time of filling and at control were used to assess outcomes by 2 different criteria (Table 2).

### 2.4. Statistical Analysis

Chi-square test was used to determine differences in treatment outcomes among groups of teeth. Actual *p* values were recorded. Logistic regression analyses of outcomes were performed for a comprehensive evaluation of all recorded factors influencing outcomes, with a sequential elimination of factors with *p* values below 0.2. All analyses were performed with Stata version 18.0 software [20].

## 3. Results

### 3.1. Characterization of Cases

Recall cases vs. cases excluded. The recorded parameters were generally similar for the no recall cases (NOC) and the recall cases (REC); however, there were more teeth with PAI score 4 and more teeth with soft tissue affections at completion of treatment in the NOC group (Supplementary Table S1).

### 3.2. Factors Influencing Treatment Outcome

Binary analyses. The success rate of treatment for all recalled teeth was 69% by strict and 84% by lenient criteria. The results of all binary analyses are given in Supplementary Table S2. PAI score at start, patients’ age, periodontal status, and the number of visits showed strong associations with the outcome, and there was a tendency for premolars and molars to do better than anterior teeth (Table 3).

While pain/discomfort had a marginal association with better outcomes for teeth with pain at the time of filling (*p* = 0.18), root fillings with questionable density tended to have better outcomes than densely filled teeth (*p* = 0.09); these subgroups had very low numbers, showed no effect in preliminary regression analyses and were therefore excluded from the final model (Supplementary Table S2A,B).

Logistic regression analyses. Logistic regression with independent variables selected from binary comparisons confirmed the preoperative PAI score, number of visits, marginal bone level, age, and tooth group as primary influences on the outcome (Table 4). Results with lenient criteria lost the effect of the PAI score, age, and bone height, but the effect of tooth type and the number of visits remained (Table 4).

### 3.3. Binary Analyses of Subgroups of Categories with Impact on the Regression

Age distribution: When cases were grouped by age as younger than 35, from 35 to 65, and above 65, the older age group (>65) contributed most to the effect of age, as shown in Table 5.

Periodontal status: Cases were grouped by bone height into four categories: >2/3 of root length; from 1/2 to 2/3 of root length; from 1/3 to 1/2 of root length; and <1/3 of root length. Seven teeth could not be scored reliably. The negative effect on prognosis was clearly greater for the latter two categories, as shown in Table 5.

Tooth group: Teeth were grouped as anterior teeth (A), premolars (P), and molars (M). Premolars tended to do better than molars and especially anterior teeth, as shown in Table 5.

Number of visits: When cases were grouped as single-visit (SV) and multiple-visit (MV) cases, the SV cases had a higher success rate (80 vs. 66%) in chi-square analyses (*p* = 0.045). When comparing SV with two visits only (TV), the association weakened but was still strong (*p* = 0.076), as shown in Table 5.

## 4. Discussion

This study focused exclusively on teeth with a radiographically confirmed diagnosis of apical periodontitis, representing a definitive sign of infection. Most of the commonly recognized tooth-related factors were registered in our study; however, patients’ health status and smoking habits were not registered. While these factors may influence the outcome to a degree [2], there is little reason to assume that they would be systematically distributed differently among the compared groups.

About 46 per cent of the cohort studied did not have adequate details or did not appear for a follow-up. These cases were largely similar to the cases with control but differed in the distribution of PAI scores in that more had PAI 3 at the start. While it may be assumed that, as a group, these teeth would therefore have had a better prognosis, this difference would not likely affect the generalizability of the main findings.

The true outcome estimate will also be influenced by factors not recorded here, such as smoking, general health issues, and the experience of the operator. However, there is no reason to believe that such factors would be distributed unevenly across the groups we have analyzed, which makes it likely that the results in binary and in regression analyses are valid for comparisons among and between groups of teeth. The type of restoration did not influence the outcome in the present study. However, only 44 teeth had a temporary filling at recall (Supplementary Table S2A).

Up to 4 years may be required to record a stable outcome that reflects the true prognosis of the endodontic treatment [21], but the general trend of healing is evident after one year [22]. Our patients were followed for up to four years, which is consistent with recommendations [18], but we chose control data closest to one year for groups to be comparable in this respect, even when later information was available. One year is also the standard recall period recommended [23].

The preoperative PAI score was another robust predictor of healing in the present study. Teeth with higher baseline scores had lower healing rates, which is consistent with the literature [5] and with our understanding that more advanced lesions require longer healing processes.

The outcome was also significantly influenced by age. Technical challenges during root canal treatment may increase with age, particularly due to the increased occurrence of root canal calcifications [24,25]. A systematic review examining longitudinal outcomes of endodontic treatment suggested that increased patient age is not generally a prognostic factor [26], but a more recent report found that younger patients tended to have more favorable outcomes [27]. Periodontal status similarly showed a strong association with outcome in our study, which is consistent with most reports on the marginal bone level and endodontic treatment outcome [2]. Given that age and marginal bone level were highly correlated, this could help explain the strong effect of age in our material. Despite this correlation, however, both factors retained a strong impact when combined in regression analyses. Therefore, it may be possible that they may differ in the way they influence the prognosis.

Tooth type also influenced the prognosis. Anterior teeth, despite generally simpler root canal anatomy, demonstrated lower success rates compared to premolars and molars in our cohort. This may be related to unmeasured clinical factors, like calcified root canals in traumatized teeth. Radiographic interpretation may be affected by anatomical variation in surrounding structures. The maxillary zygomatic process and the dense cortical bone in the lower jaw may obscure early periapical radiolucencies.

The results of this study demonstrated that the radiographic outcome was negatively affected by the number of visits, even when corrected for confounders in regression analyses.

The time and visits needed to treat chronic apical periodontitis in a practice setting depends on numerous factors. Available time and case complexity are important factors, and simple cases that may be treated quickly are obviously more often treated in a single visit. Moreover, the present study was conducted in a teaching environment where didactic considerations may have played a role, thus making the assignment of cases to one or more visits even more complex. However, single- vs. multiple-visit approaches may differ in a way that could be reflected in the outcome irrespective of the mode of case selection for the two approaches: on the one hand, instrumentation and disinfection in one appointment may not be sufficient for adequate disinfection, thereby necessitating and benefitting from more appointments; on the other, inadequacies in instrumentation, irrigation, medication, and the temporary filling may impair rather than improve infection control in two or more appointments.

The results were conspicuously poorer for teeth that were treated in more than three sessions: these teeth could be complex with a poor prognosis from the outset regardless of the number of visits. We therefore did a separate analysis comparing single-visit cases with cases completed in two sessions, which would exclude many such cases that would otherwise reduce the success rate of multi-visit cases. While the difference in favor of single-visit treatment was reduced when cases with more than two visits were excluded, the association was still strong. These findings strengthen the clinical practice guidelines from The European Society of Endodontology, which recommend using a single-visit approach without the use of interappointment dressing with Ca(OH)_2_ in cases where appropriate clinical procedures can be performed within an adequate time frame [18,23].

## 5. Conclusions

The overall outcome of the first-time treatment of teeth with periapical lesions by postgraduate students was negatively influenced by a higher preoperative PAI scores, higher number of visits, poorer periodontal status, older age, and anterior tooth type. Single-visit treatment showed better outcomes compared to multiple- or two-visit treatment in binary and regression analyses, suggesting that a single-visit approach, when it is clinically appropriate, may offer higher radiographic success and boost efficiency in a postgraduate endodontic setting.

## Figures and Tables

**Table 1:** Data recorded for each tooth used in the analyses.

Patient Factors
Age and sex
Tooth-related factors
Tooth type
Bone level relative to root length
Any pain or discomfort associated with the tooth
Swelling or tenderness of soft tissue
Percussion sensitivity
Complications during treatment
PAI score at time of filling
PAI score at time of control
Procedural factors
Number of visits to completion
Root filling homogeneity
Root filling length; surplus

Details in Supplementary Tables.

**Table 2:** Outcome measures.

Target Endpoints
Strict criteria: Healing: PAI at control 1 or 2. Not healing: PAI at control 3, 4, or 5.
Lenient criteria: Healing: PAI at control = 1 or 2 and PAI at control = 3 for teeth with PAI = 4 or 5 at start. Not healing: PAI = 3 both at completion of treatment and at control, and all PAI = 4 or 5 at control.

**Table 3:** Factors influencing outcomes in binary analyses.

Factor	Trend	*p* Value Strict	*p* Value Lenient
PAI at start	Lower PAI at start, better outcome	<0.001	0.644
No. of visits	Fewer visits, better outcome	0.020	0.142
Age	Younger patients, better outcome	0.020	0.435
Periodontal status	Poorer status, poorer outcome	0.024	0.474
Tooth type	Premolars and molars, better outcome	0.093	0.006

**Table 4:** Logistic regression analyses of relevant factors influencing outcomes.

Strict criteria						
**Success**	**Odds Ratio**	**Std. Err.**	**z**	**p > z**	**95% Conf. Interval**	
PAI at start	1.937	0.302	4.24	<0.001	1.43	2.63
No. of visits	1.325	0.127	2.94	0.003	1.1	1.6
Age	1.011	0.006	2.03	0.042	1	1.02
Periodontal score	1.329	0.18	2.1	0.036	1.02	1.73
Tooth type	0.648	0.159	−1.77	0.077	0.4	1.05
Total *p* value < 0.0001						
Pseudo R square = 0.0795			*n* = 430			
Lenient criteria						
**Success**	**Odds Ratio**	**Std. Err.**	**z**	**p > z**	**95% Conf. Interval**	
PAI at start	0.802	0.154	−1.15	0.249	0.55	1.17
No. of visits	1.253	0.134	2.12	0.034	1.02	1.55
Age	1.006	0.007	0.97	0.334	0.99	1.02
Periodontal score	1.213	0.192	1.22	0.222	0.89	1.65
Tooth type	0.403	0.114	−3.21	0.001	0.23	0.70
Total *p* value = 0.0091						
Pseudo R square = 0.0388			*n* = 430			

**Table 5:** Binary analyses of treatment success for groups with impact in regression. Strict criteria; percent distributions.

**Age**						
*p* = 0.020		<35 yrs	35–65 yrs	>65 yrs	All	
	Healed	73.94	68.72	57.76	67.51	
	Not healed	26.06	31.28	42.24	32.49	
	Total no.	142	179	116	437	
						
**B one height**						
*p* = 0.024		>2/3	1/2–2/3	1/3–1/2	<1/3	All
	Healed	70.48	64.38	44.00	52.94	67.21
	Not healed	29.52	35.62	56.00	47.06	32.79
	Total no. ^1^	315	73	25	17	430
**Tooth group**						
*p* = 0.201		Anterior	Premolar	Molar	All	
	Healed	61.34	72.86	68.95	67.51	
	Not healed	38.66	27.14	31.05	32.49	
	Total no.	119	70	248	437	
						
**Single visit vs. multiple visits**						
*p* = 0.045		Single v	Multiple v	All		
	Healed	80.00	65.89	67.51		
	Not healed	20.00	34.11	32.49		
	Total no.	50	387	437		
						
**Single visit vs. two visits**						
*p* = 0.076		Single v	Two v	All		
	Healed	80.00	67.26	69.57		
	Not healed	20.00	32.74	30.43		
	Total no.	50	226	276		

^1^ Seven cases were not scored for bone height.

## Data Availability

The raw data supporting the conclusions of this article will be made available by the authors on request.

## Supplementary Materials

The following supporting information can be downloaded at: https://www.mdpi.com/article/10.3390/dj13120593/s1, Table S1. Comparison of recalled cases (REC) with cases with inadequate or no control data (NOC). Distributions in percent. Table S2. Associations of outcome with recorded variables; chi-square analyses, (A) strict criteria, and (B) lenient criteria.

## References

[1] Piñas-Alonzo R., Bello R., Hernández A., Lacerda M., Vinuesa-Maqueda C., Pérez-Ron O., Graterol I.G., Pérez A.R. (2025). Clinical outcomes and prognostic factors in endodontic treatment: A systematic review from 2002–2022. Br. Dent. J..

[2] Kirkevang L.L., Vaeth M., Orstavik D. (2020). Epidemiology, Treatment Outcome, and Risk Factors for Apical Periodontitis. Essential Endodontology: Prevention and Treatment of Apical Periodontitis.

[3] Al Jallad N., Sun E., Wu T., Cui S., Basmaji A., Thakkar R., Aboelmagd S., Naik N., Tzouma K., Xiao J. (2025). The Success of Endodontic Treatments Performed by Dental Residents in Advanced Education in General Dentistry Program: A 10-Year Retrospective Study. Dent. J..

[4] Khandelwal A., Janani K., Teja K., Jose J., Battineni G., Riccitiello F., Valletta A., Palanivelu A., Spagnuolo G. (2022). Periapical Healing following Root Canal Treatment Using Different Endodontic Sealers: A Systematic Review. Biomed. Res. Int..

[5] Kirkevang L.L., Orstavik D., Wenzel A., Vaeth M. (2014). Prognostic value of the full-scale Periapical Index. Int. Endod. J..

[6] Paredes-Vieyra J., Enriquez F.J. (2012). Success rate of single- versus two-visit root canal treatment of teeth with apical periodontitis: A randomized controlled trial. J. Endod..

[7] Karaoğlan F., Miçooğulları Kurt S., Çalışkan M.K. (2022). Outcome of single- versus two-visit root canal retreatment in teeth with periapical lesions: A randomized clinical trial. Int. Endod. J..

[8] Moreira M.S., Anuar A.S.N.-S., Tedesco T.K., Dos Santos M., Morimoto S. (2017). Endodontic treatment in single and multiple visits: An overview of systematic reviews. J. Endod..

[9] Schwendicke F., Gostemeyer G. (2017). Single-visit or multiple-visit root canal treatment: Systematic review, meta-analysis and trial sequential analysis. BMJ Open.

[10] Mergoni G., Ganim M., Lodi G., Figini L., Gagliani M., Manfredi M. (2022). Single versus multiple visits for endodontic treatment of permanent teeth. Cochrane Database Syst. Rev..

[11] Rossi-Fedele G., Roedig T. (2023). Effectiveness of root canal irrigation and dressing for the treatment of apical periodontitis: A systematic review and meta-analysis of clinical trials. Int. Endod. J..

[12] Chung S.H., Chang J. (2021). Impact of endodontic case difficulty on operating time of single visit nonsurgical endodontic treatment under general anesthesia. BMC Oral Health.

[13] Peters L.B., van Winkelhoff A.J., Buijs J.F., Wesselink P.R. (2002). Effects of instrumentation, irrigation and dressing with calcium hydroxide on infection in pulpless teeth with periapical bone lesions. Int. Endod. J..

[14] Zandi H., Kristoffersen A.K., Orstavik D., Rocas I.N., Siqueira J.F., Enersen M. (2018). Microbial Analysis of Endodontic Infections in Root-filled Teeth with Apical Periodontitis before and after Irrigation Using Pyrosequencing. J. Endod..

[15] Jurič R., Vidmar G., Blagus R., Jan J. (2024). Factors associated with the outcome of root canal treatment—A cohort study conducted in a private practice. Int. Endod. J..

[16] Field J.W., Gutmann J.L., Solomon E.S., Rakusin H. (2004). A clinical radiographic retrospective assessment of the success rate of single-visit root canal treatment. Int. Endod. J..

[17] Orstavik D., Kerekes K., Eriksen H.M. (1986). The periapical index: A scoring system for radiographic assessment of apical periodontitis. Endod. Dent. Traumatol..

[18] European Society of Endodontology (2006). Quality guidelines for endodontic treatment: Consensus report of the European Society of Endodontology. Int. Endod. J..

[19] Landis J.R., Koch G.G. (1977). The measurement of observer agreement for categorical data. Biometrics.

[20] Stata (2023). Stata Statistical Software: Release 18.

[21] Kerekes K., Tronstad L. (1979). Long-term results of endodontic treatment performed with a standardized technique. J. Endod..

[22] Orstavik D. (1996). Time-course and risk analyses of the development and healing of chronic apical periodontitis in man. Int. Endod. J..

[23] Duncan H.F., Kirkevang L.L., Peters O.A., El-Karim I., Krastl G., Del Fabbro M., Chong B.S., Galler K.M., Segura-Egea J.J., Kebschull M. (2023). Treatment of pulpal and apical disease: The European Society of Endodontology (ESE) S3-level clinical practice guideline. Int. Endod. J..

[24] Morse D.R., Esposito J.V., Schoor R.S., Williams F.L., Furst M.L. (1991). A review of aging of dental components and a retrospective radiographic study of aging of the dental pulp and dentin in normal teeth. Quintessence Int..

[25] Kiefner P., Connert T., ElAyouti A., Weiger R. (2017). Treatment of calcified root canals in elderly people: A clinical study about the accessibility, the time needed and the outcome with a three-year follow-up. Gerodontology.

[26] Shakiba B., Hamedy R., Pak J.G., Barbizam J.V., Ogawa R., White S.N. (2017). Influence of increased patient age on longitudinal outcomes of root canal treatment: A systematic review. Gerodontology.

[27] Liu S.-Q., Chen X., Wang X.-X., Liu W., Zhou X., Wang X. (2021). Outcomes and prognostic factors of apical periodontitis by root canal treatment and endodontic microsurgery—A retrospective cohort study. Ann. Palliat. Med..

