# Supplementary material for: Radiographic Outcome of Endodontic Treatment of Teeth with Primary Apical Periodontitis: Results from a Postgraduate Clinic
Source: Dent J (Basel). 2025 Dec 11;13(12):593. doi: 10.3390/dj13120593 (PMC12731401; doi:10.3390/dj13120593)
Supplement: Supplementary file 1 [file dentistry-13-00593-s001.zip › dentistry-3933239-supplementary.pdf]

Supplementary Table S1. Comparison of recalled cases (REC) with cases with inadequate or no control data (NOC). Distributions in per cent.

		NOC group N=367	REC group N=437	N	p value
Sex				804	0.774
	Female	52.04	51.03	414	
	Male	47.96	48.97	390	
Age				804	0.187
	<35 yrs	31.06	32.49	256	
	35-65 yrs	46.87	40.96	351	
	>65 yrs	22.07	26.54	197	
PAI at start				804	0.001
	PAI3	28.88	37.07	268	
	PAI4	58.86	46.00	417	
	PAI5	12.26	16.93	119	
Tooth group				804	0.305
	Anteriors	30.52	27.23	231	
	Premolars&Molars	69.48	72.77	573	
No of visits				804	0.658
	One	13.08	11.44	98	
	Two	47.96	51.72	402	
	Three	25.07	21.51	186	
	Four	8.99	9.61	75	
	Five+	4.90	5.72	43	
Complications during treatment				804	0.561
	None	77.93	77.57	625	
	Perforation	3.54	3.66	29	
	Instrument fracture	1.36	3.43	20	
	Exacerbation	1.36	0.69	8	
	Surplus	10.35	9.84	81	
	Inhomogenous root filling	0.82	0.46	5	
	Short	4.63	4.35	36	
Soft tissue at completion[1]				776	0.031
	Healthy	88.70	93.13	707	
	Tender	3.67	2.84	25	
	Tender and mucosal redness	1.98	1.42	13	
	Swelling	0.85	1.42	9	
	Sinus tract	4.80	1.18	22	
Percussion sensitivity at completion				804	0.165
	No	90.19	92.91	737	
	Yes	9.81	7.09	67	
Pain sensation at completion[1]				804	0.236
	None	94.82	97.25	773	
	Hot/cold	0.27	0.00	1	
	Tender on chewing	3.81	1.83	22	
	Spontaneous	1.09	0.92	8	
Root filling density at completion1				800	0.676
	Adequate	96.99	97.47	778	
	Some voids/slits	3.01	2.53	22	
Root filling length1				684	0.737
	0-2.5 mm from root apex	87.00	88.28		
	Shorter	4.67	4.95		
	Surplus	8.33	6.77		
Periodontal bone level at completion[1,2]				786	0.750
	>2/3	75.56	73.26	584	
	1/2-2/3	14.89	16.98	126	
	1/3-1/2	6.46	5.81	48	
	<1/3	3.09	3.95	28	

[1]Some cases lacked information on this item.

[2]Bone height relative to root length, subjectively assessed in radiograph

Supplementary Table S2A. Associations of outcome with recorded variables; chi square analyses. Strict criteria

		Success	Not healed	N	P value
All cases		67.51	32.49	437	
Sex				437	0.925
	Female	67.71	32.29	223	
	Male	67.29	32.71	214	
Age				437	0.020
	<35 yrs	73.94	26.06	142	
	35-65 yrs	68.72	31.28	179	
	>65 yrs	57.76	42.24	116	
PAI at start				437	<0.001
	PAI3	80.86	19.14	162	
	PAI4	62.19	37.81	201	
	PAI5	52.70	47.30	74	
Tooth group				437	0.093
	Anterior	61.34	38.66	119	
	Premolars & molars	69.81	30.19	318	
No of visits					0.020
	One	80.00	20.00	50	
	Two	67.26	32.74	226	
	Three	72.34	27.66	94	
	Four	52.38	47.62	42	
	Five and more	52.00	48.00	25	
Jaw				437	0.283
	Mandibular	64.61	35.39	178	
	Maxillary	69.50	30.50	259	
Complications during treatment				437	0.844
	None	68.14	31.86	339	
	Perforation	68.75	31.25	16	
	Instrument fracture	66.67	33.33	15	
	Exacerbation	100.00	0.00	3	
	Surplus	65.12	34.88	43	
	Inhomogenous root filling	50.00	50.00	2	
	Short	57.89	42.11	19	
Soft Tissue status at completion[1]				422	0.826
	Healthy	67.43	32.57	393	
	Tender	75.00	25.00	12	
	Tender and mucosal redness	66.67	33.33	6	
	Swelling	50.00	50.00	6	
	Sinus tract	80.00	20.00	5	
Percussion sensitivity at completion				437	0.669
	No	67.24	32.76	406	
	Yes	70.97	29.03	31	
Pain sensation at completion				437	0.176
	None	66.82	33.18	425	
	Tender on chewing	87.50	12.50	8	
	Spontaneous	100.00	0.00	4	
Root filling density at completion[1]				435	0.094
	Adequate	66.98	33.02	424	
	Some voids/slits	90.91	9.09	11	
Root filling length[1]				384	0.965
	0-2.5 mm from root apex	67.85	32.15	330	
	Shorter	68.42	31.58	19	
	Surplus	65.38	34.62	26	
Periodontal bone level at completion[2]				430	0.024
	>2/3	76.82	65.96	315	
	1/2-2/3	16.26	18.44	73	
	1/3-1/2	3.81	9.93	25	
	<1/3	3.11	5.67	17	
Restoration type at control[1]				395	0.499
	Temporary	72.73	27.27	44	
	Permanent filling	69.27	30.73	205	
	Crown	67.29	32.71	107	
	Bridge abutment	54.55	45.45	33	
	Post in canal	66.67	33.33	6	

[1]Some cases lacked information on this item.

[2]Bone height relative to root length, subjectively assessed in radiograph

Supplementary Table S2B. Associations of outcome with recorded variables; chi square analyses. Lenient criteria

		Success	Not healed	N	P value
All cases		82.84	17.16	437	
Sex				437	0.945
	Female	82.96	17.04	223	
	Male	82.71	17.29	214	
Age				437	0.435
	<35 yrs	85.92	14.08	142	
	35-65 yrs	80.45	19.55	179	
	>65 yrs	82.76	17.24	116	
PAI at start				437	0.644
	PAI3	80.86	19.14	162	
	PAI4	84.58	15.42	201	
	PAI5	82.43	17.57	74	
Tooth group				437	0.006
	Anterior	74.79	25.21	119	
	Premolars & molars	85.85	14.15	318	
No of visits					0.142
	One	88	12	50	
	Two	82.74	17.26	226	
	Three	87.23	12.77	94	
	Four	71.43	28.57	42	
	Five and more	76.00	24.00	25	
Jaw				437	0.510
	Mandibular	84.27	15.73	178	
	Maxillary	81.85	18.15	259	
Time to control				437	0.374
	<18 months	78.67	83.98	367	
	18-36 months	16.00	12.36	54	
	>36 months	5.33	3.66	16	
Complications during treatment				437	0.547
	None	83.48	16.52	339	
	Perforation	93.75	6.25	16	
	Instrument fracture	73.33	26.67	15	
	Exacerbation	100	0	3	
	Surplus	79.07	20.93	43	
	Inhomogenous root filling	100	0	2	
	Shortroot filling	73.68	26.32	19	
Soft tissue status at completion[1]				422	0.417
	Healthy	82.95	17.05	393	
	Tender	91.67	8.33	12	
	Tender and mucosal redness	66.67	33.33	6	
	Swelling	66.67	33.33	6	
	Sinus tract	100	0	5	
Percussion sensitivity at completion				437	0.737
	No	83	17	406	
	Yes	80.65	19.35	31	
Pain sensation at completion				437	0.616
	None	82.59	17.41	425	
	Tender on chewing	87.5	12.5	8	
	Spontaneous	100.00	0.00	4	
Root filling density at completion[1]				435	0.128
	Adequate	82.55	17.45	424	
	Some voids/slits	100	0	11	
Root filling length[1]				384	0.776
	0-2.5 mm from root apex	84.07	15.93	330	
	Shorter	78.95	21.05	19	
	Surplus	80.77	19.23	26	
Periodontal bone level at completion[1,2]				430	0.474
	>2/3	74.44	67.57	315	
	1/2-2/3	16.57	18.92	73	
	1/3-1/2	5.62	6.76	25	
	<1/3	3.37	6.76	17	
Restoration type at control[1]				395	0.193
	Temporary	84.09	15.91	44	
	Permanent filling	82.93	17.07	205	
	Crown	85.98	14.02	107	
	Bridge abutment	69.7	30.3	33	
	Post in canal	100	0	6	

[1]Some cases lacked information on this item.

[2]Bone height relative to root length, subjectively assessed in radiograph
